# The polo-like kinase 1 inhibitor volasertib synergistically increases radiation efficacy in glioma stem cells

**DOI:** 10.18632/oncotarget.24041

**Published:** 2018-01-08

**Authors:** Jianwen Dong, Soon Young Park, Nghi Nguyen, Ravesanker Ezhilarasan, Emmanuel Martinez-Ledesma, Shaofang Wu, Verlene Henry, Yuji Piao, Ningyi Tiao, David Brunell, Clifford Stephan, Roel Verhaak, Erik Sulman, Veerakumar Balasubramaniyan, John F. de Groot

**Affiliations:** ^1^ Department of Neuro-Oncology, The University of Texas MD Anderson Cancer Center, Houston, TX, USA; ^2^ Institute of Biosciences and Technology, Texas A&M Health Science Center at Houston, Center for Translational Cancer Research, Houston, TX, USA; ^3^ Division of Radiation Oncology, The University of Texas MD Anderson Cancer Center, Houston, TX, USA; ^4^ Department of Bioinformatics and Computational Biology, The University of Texas MD Anderson Cancer Center, Houston, TX, USA; ^5^ Department of Genomic Medicine, The University of Texas MD Anderson Cancer Center, Houston, TX, USA; ^6^ The Jackson Laboratory for Genomic Medicine, Farmington, CT, USA

**Keywords:** glioblastoma, polo-like kinase 1, volasertib, radiation, sensitization

## Abstract

**Background:**

Despite the availability of hundreds of cancer drugs, there is insufficient data on the efficacy of these drugs on the extremely heterogeneous tumor cell populations of glioblastoma (GBM).

**Results:**

The PKIS of 357 compounds was initially evaluated in 15 different GSC lines which then led to a more focused screening of the 21 most highly active compounds in 11 unique GSC lines using HTS screening for cell viability. We further validated the HTS result with the second-generation PLK1 inhibitor volasertib as a single agent and in combination with ionizing radiation (IR). *In vitro* studies showed that volasertib inhibited cell viability, and high levels of the anti-apoptotic protein Bcl-xL expression were highly correlated with volasertib resistance. Volasertib sensitized GSCs to radiation therapy by enhancing G2/M arrest and by inducing apoptosis. Colony-formation assay demonstrated that volasertib plus IR synergistically inhibited colony formation. In intracranial xenograft mouse models, the combination of volasertib and radiation significantly inhibited GSC tumor growth and prolonged median survival compared with radiation treatment alone due to inhibition of cell proliferation, enhancement of DNA damage, and induction of apoptosis.

**Conclusions:**

Our results reinforce the potential therapeutic efficacy of volasertib in combination with radiation for the treatment of GBM.

**Methods:**

We used high-throughput screening (HTS) to identify drugs, out of 357 compounds in the published Protein Kinase Inhibitor Set, with the greatest efficacy against a panel of glioma stem cells (GSCs), which are representative of the classic cancer genome atlas (TCGA) molecular subtypes.

## INTRODUCTION

Glioblastoma (GBM) is the most common and aggressive form of primary brain tumors in adults. Current therapy for GBM, using a combination of surgery, radiation, and chemotherapy, reduces intracranial tumor burden with modest efficacy in prolonging survival [1]. Patients with GBM have a mean survival of 12 to 14 months from the time of diagnosis, with fewer than 5% of patients alive at 3 years [1, 2]. Despite the availability of hundreds of cancer drugs, there are limited treatment options for patients, and there is insufficient information on the efficacy of these drugs in the extremely heterogeneous populations of tumor cells in GBM [3].

GBM displays striking intratumoral heterogeneity and a high resistance to radiation and chemotherapy, due to the presence of stem-like glioma stem cells (GSCs), also called glioma tumor-initiating cells (GSCs). GSCs have the ability to undergo self-renewal and initiate tumorigenesis, and they are resistant to a wide variety of chemotherapeutic agents and possess a remarkable ability to recover from cytotoxic therapy [4, 5]. The advent of high-throughput screening (HTS) has enabled the screening of large, diverse compound libraries against a panel of cells to validate targets and identify drug candidates for clinical development [6]. Therefore, integration of comprehensive HTS of molecularly targeted agents with patient-derived GSCs that have been extensively profiled by multiple “omics” techniques, such as genomics, methylomics and proteomics, provides an extraordinary opportunity to develop targeted therapies for subsets of patients with GBM [7]. In this study, HTS was used to identify drug sensitivities to 357 compounds in the published Protein Kinase Inhibitor Set (PKIS) from GlaxoSmithKline (GSK) using a panel of GSCs, which are representative of the classic cancer genome atlas (TCGA) molecular subtypes [8] and are highly characteristic of human glioma growth patterns that contribute to tumor initiation and therapeutic resistance. From the initial 357 compounds, the 21 most highly active compounds were more extensively studied. This HTS screen identified sensitivity of GSCs to inhibitors of polo-like kinase-1 (PLK1), a key regulator of mitosis [9]. Given that PLK1 is often overexpressed in a broad spectrum of cancers, with highest expression levels being correlated with poor prognosis in several cancer types [10–13], we further validated the HTS result with the second-generation PLK1 inhibitor volasertib (BI6727, Boehringer Ingelheim, Germany) as a single agent and in combination with ionizing radiation (IR). Here, we describe, for the first time, the *in vitro* and *in vivo* efficacy of volasertib as a single agent and combined with radiation in GSCs.

## RESULTS

### High-throughput screening of published PKIS compounds against GSCs identifies PLK1 as a potential therapeutic target for glioblastoma

**Figure 1:**
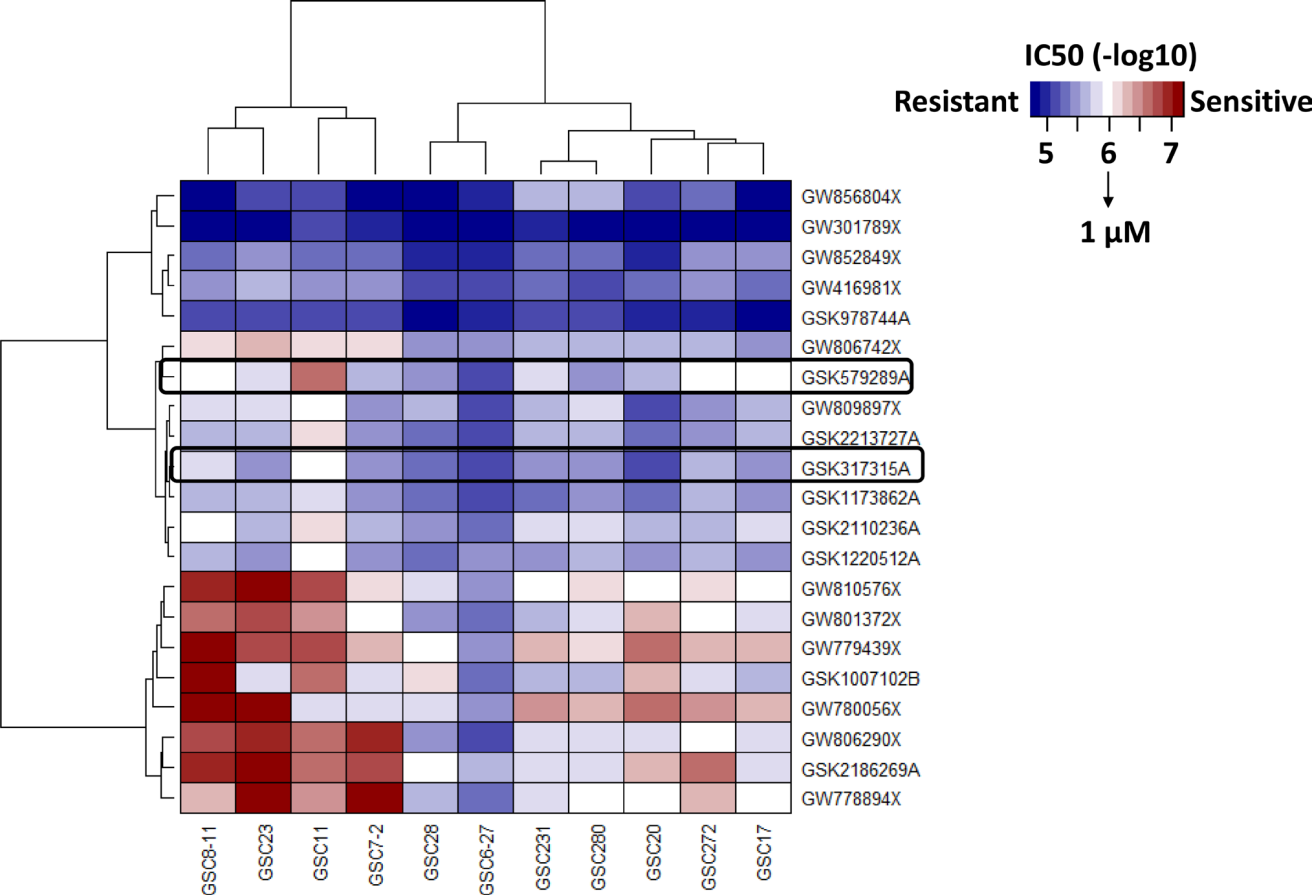
An HTS assay was used to identity drug sensitivities of 21 compounds in the PKIS (GSK) in a panel of 11 GSC lines, which were representative of the classic cancer genome atlas (TCGA) molecular subtypes Heatmap, depicting the IC50 of these compounds, identified PLK1 as a therapeutic target of GSC.

Two PKIS compound libraries were obtained from GlaxoSmithKline, and both compound libraries have been tested for kinase activity. The compound structure and PKIS data are readily available at ChEMBL [14, 15]. The PKIS of 357 compounds was initially evaluated on 15 different cell lines of GSCs (data not shown) which led to a more focused screening of the 21 most highly active compounds in 11 unique cell lines of GSCs (Supplementary Table 1). We used an IC_50_ of < 1 μM as a cutoff for sensitivity of each compound. We used the IC_50_ cutoff values given that they demonstrated a good correlation with area under the curve (AUC) and because IC_50_ provides a dose reference for further *in vitro* and *in vivo* experiments (Supplementary Table 2). The heatmap depicting the IC_50_ of 21 compounds against the 11 GSC lines is shown in Figure 1. Of these 21 compounds, we found 8 compounds with potent inhibition of cell viability in at least 10 GSC lines; however, some of these compounds demonstrated potent activity at nanomolar concentrations against a large number of kinases, creating challenges in determining mechanism of action. In contrast, 10 compounds showed little or no cytotoxicity against at least 10 GSC lines. Furthermore, only two compounds (GSK978744A and GW301789X) showed statistically significant association to TCGA subtypes (Supplementary Table 3) but without having any GSCs sensitive to these compounds. Interestingly, our HTS data showed that 2 GSC lines had IC_50_ < 1 μM, 8 GSC lines had IC_50_ ranging from 1 to 5 μM, and 1 GSC line had IC_50_ > 5 μM for GSK579289A; whereas for GSK317315A, 1 GSC line had IC_50_ < 1 μM, 8 GSC lines had IC_50_ ranging from 1 to 5 μM, and 2 GSC lines had IC_50_ > 5 μM. GSK579289A and GSK317315A have been tested for kinase inhibitory activity, and both selectively and potently inhibit PLK1 (96% inhibition for GSK579289A, 97% inhibition for GSK317315A) at a low concentration (100 nM) [14, 15]. In summary, our HTS assay of PKIS compounds against a panel of GSC lines indicated that PLK1 is a potential therapeutic target of GBM.

### Inhibition of PLK1 by volasertib results in cell cycle arrest and apoptosis in GSCs

**Figure 2:**
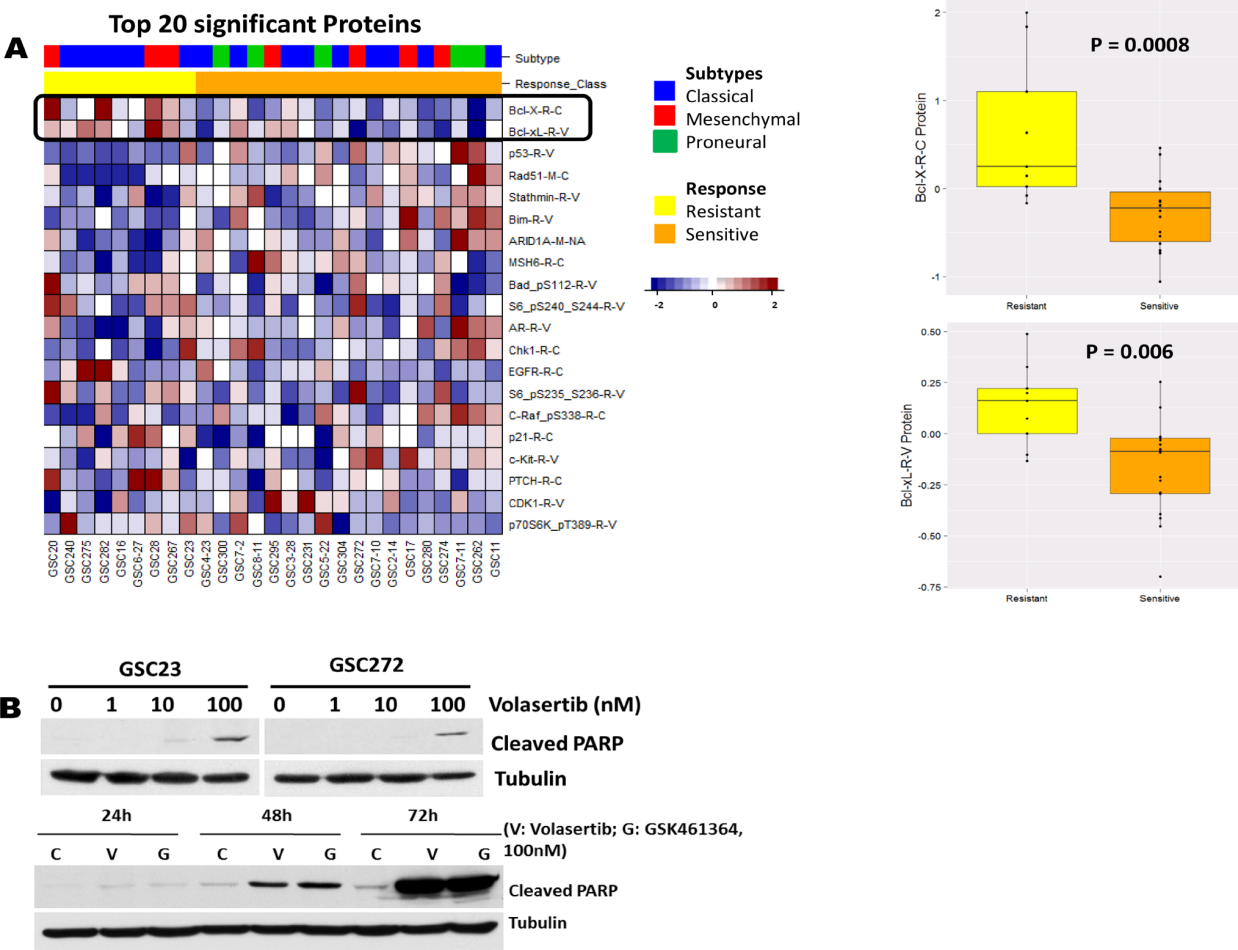
The HTS result was further verified *in vitro* with the second-generation PLK1 inhibitor volasertib (**A**) Heatmap from the top ranked 20 proteins associated to Volasertib sensitivity; anti-apoptotic Bcl-2 family proteins were the most significant. Correlation of the volasertib IC_50_ with fold-change (log2) in protein expression in 27 GSC lines demonstrated that high expression levels of anti-apoptotic Bcl-2 family proteins were a predictor of resistance to volasertib. (**B**) Whole-cell protein extracts were analyzed after different time points of treatment with different concentrations of volasertib and GSK461364 by Western blot, with the indicated antibodies. Representative Western blot data demonstrated that volasertib induced prominent c-PARP in a dose- and time-dependent manner, indicating that inhibition of PLK1 resulted in apoptosis in a time- and dose-dependent manner.

PLK1 is often overexpressed in a broad spectrum of cancers, including GBM (proteinatlas.org), and high PLK1 expression levels correlate with poor prognosis. We further verified our HTS results by testing the second-generation PLK1 inhibitor volasertib on GSCs. We tested 27 GSC lines to determine their sensitivity to volasertib and found that volasertib inhibited cell viability with an IC_50_ ranging from 7.72 nM to 11.4 μM (data not shown). Moreover, we correlated proteomic RPPA data with volasertib responses of 27 GSC subtypes to find predictors of drug sensitivity. Figure 2A shows a heatmap from the top 20 significant proteins correlated to volasertib sensitivity based on a distinct protein expression pattern among sensitive and resistant GSCs. Among these proteins, high expression of anti-apoptotic Bcl-2 family protein was highly correlated with volasertib resistance (*P* < 0.00033, Figure 2A), suggesting apoptosis contributed to the antitumor activity of volasertib. Consistent with this finding, treatment with volasertib induced prominent poly ADP ribose polymerase cleavage (c-PARP) in a dose-dependent and time-dependent manner (Figure 2B), suggesting volasterib treatment led to substantial induction of apoptosis in GSCs. In addition, Supplementary Figure 1 shows that Volasertib response has not association with a specific TCGA subtype indicating that Volasertib is a good candidate for clinical trials independent of tumor subtype.

PLK1 is a critical regulator of cell cycle progression [13, 16]. To investigate the effects of volasertib on GSC cell cycle distribution, we subjected GSCs to three different concentrations (1 nM, 10 nM, and 100 nM) of volasertib for different times (16, 24, and 48 hours) and then, measured the cell cycle by flow cytometry (Supplementary Figure 2). We observed a dose- and time-dependent increase in cells in G2/M phase, as well as a sub-G1 accumulation after longer exposure to volasertib, indicating that volasertib abrogates mitosis followed by induction of apoptosis. These data are consistent with our results showing volasertib-mediated PARP cleavage and results observed by others in HeLa cells, HUVECs and NSCLC [17].

### PLK1 inhibition by volasertib enhances the radiosensitivity of GSCs by modulating cell cycle arrest

**Figure 3:**
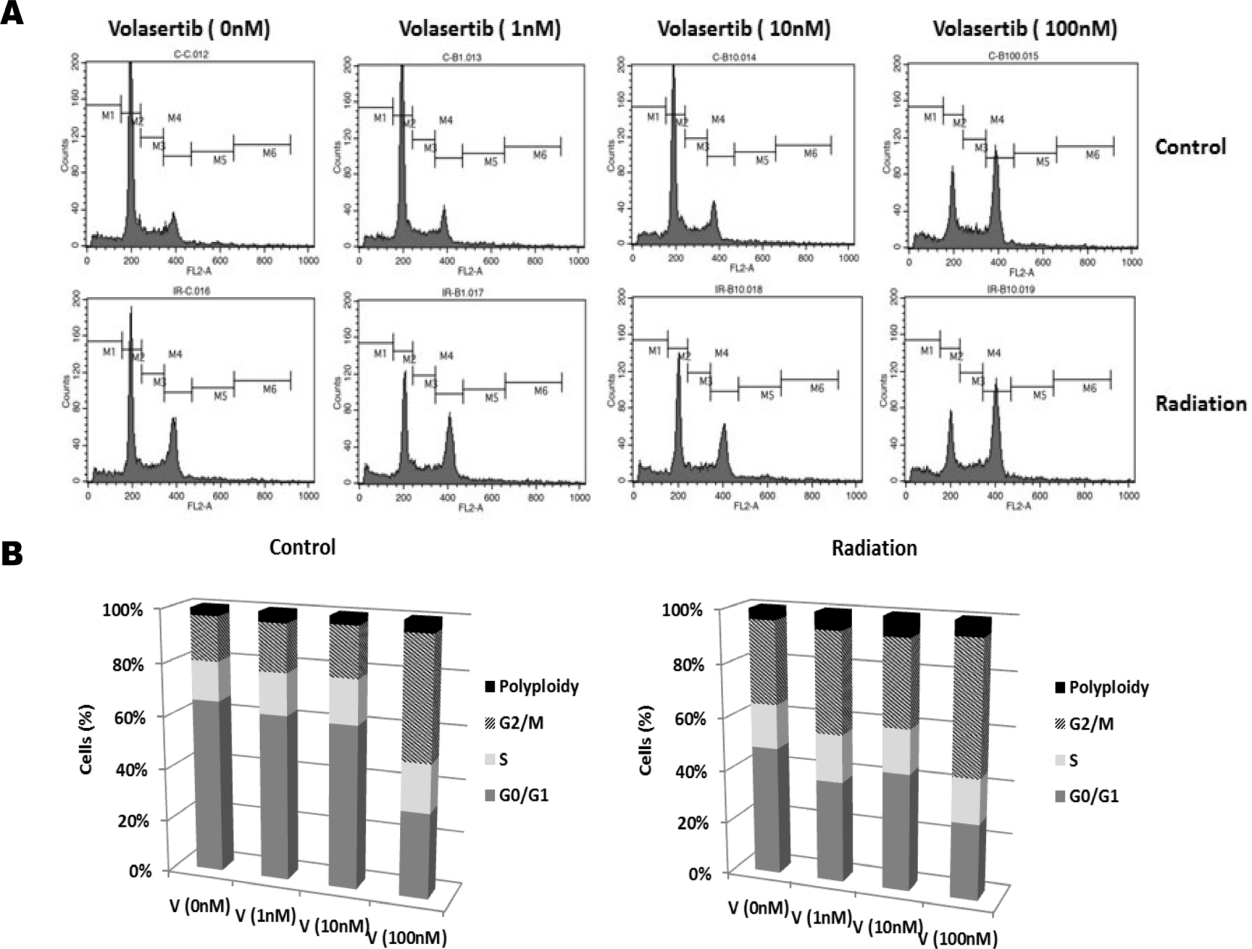
G2/M arrest induced by volasertib was additively enhanced by radiation GSCs were subjected to 2 Gy of ionizing radiation after 24 hours of treatment with volasertib, and then cell cycle distribution was analyzed 48 hours after radiation. Representative (**A**) and group (**B**) data of cell cycle distribution demonstrated that volasertib and radiation had an additive effect on G2/M arrest, in a dose-dependent manner. Shown are representative data from three individual experiments.

Given previous findings that radiosensitivity of cells is dependent on the phase of the cell cycle [18, 19], with cells in S phase being the most radioresistant and cells in G2/M the most radiosensitive, we examined the combined effect of volasertib and radiation on cell cycle arrest. Representative and group data of cell cycle distribution, shown in Figure 3, demonstrated that inhibition of PLK1 with volasertib resulted in a dose-dependent G2/M arrest and polyploidy production in GSCs. Polyploidy can be induced by persistent DNA damage signaling [20, 21], and cells unable to undergo mitosis (i.e, mitotic catastrophe) can demonstrate polyploidy. Therefore, our data suggest that volasertib led to DNA damage and to induction of mitotic catastrophe in GSCs. Combining volasertib with 2 Gy of radiation additively enhanced G2/M arrest and polyploidy production. These findings are consistent with previous reports showing that PLK1 inhibition with GSK461364A in U87 and U251 GBM [22] and with BI2536 in medulloblastoma cells [23] induced mitotic catastrophe and enhanced radiosensitivity.

### Volasertib synergistically enhances the sensitivity to radiation by apoptosis induction, DNA damage, and inhibition of colony formation

**Figure 4:**
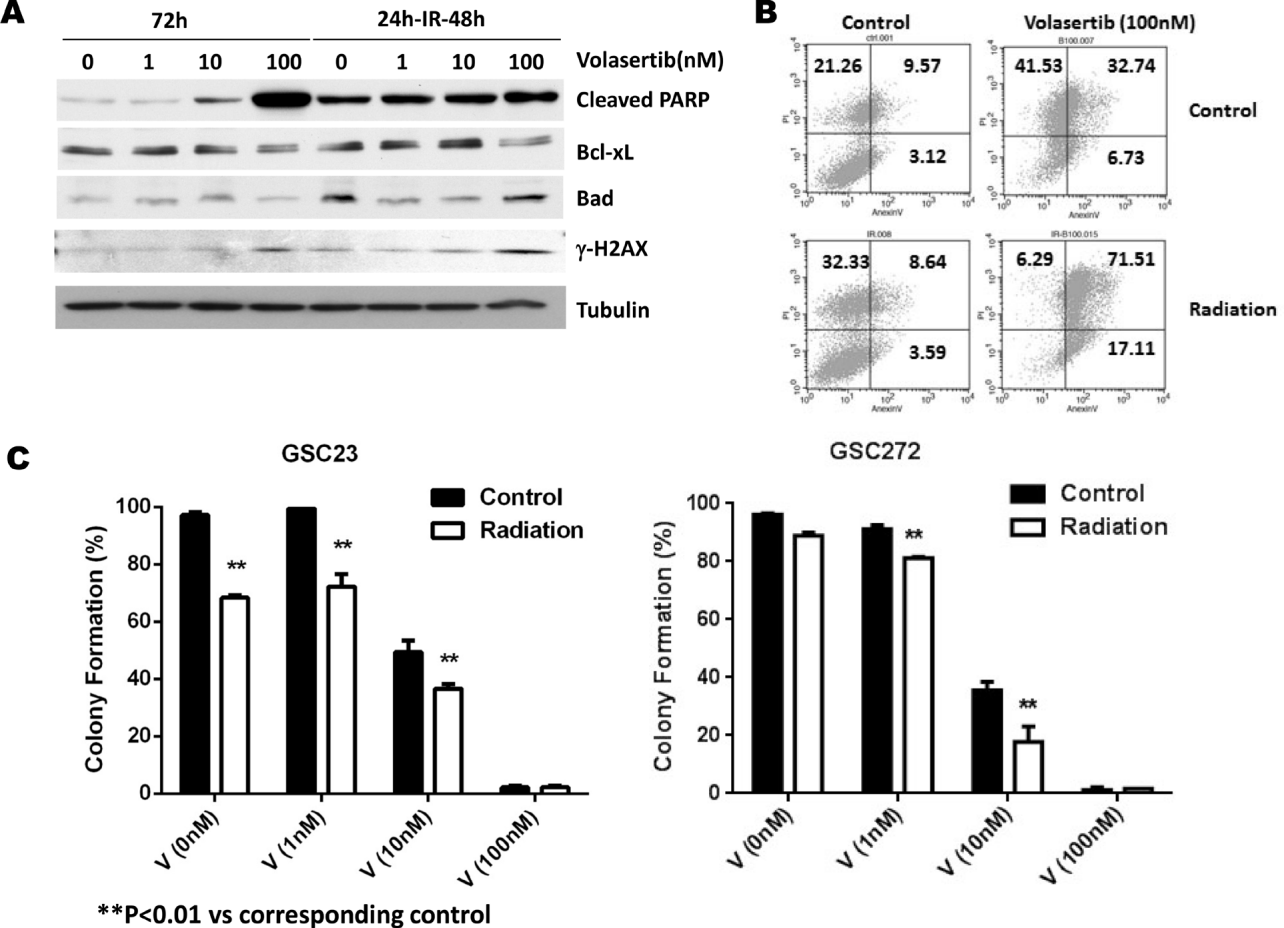
Effects of PLK1 inhibition by volasertib and radiation on apoptosis induction, cell cycle arrest, and clonogenic assay for self-renewal of GSCs (**A**) Western blotting data showed prominent PARP cleavage in the combination treatment group compared with the volasertib treatment alone group. γ-H2AX and cyclin B1 expression levels were higher in the combination treatment group than in the volasertib-treatment alone group. (**B**) Flow cytometric assay of apoptosis by annexin V staining confirmed increased apoptosis induction in response to combination treatment than to single treatment. (**C**) Colony-formation assay demonstrated that volasertib combined with radiation had a synergistic effect on inhibiting colony formation in GSCs. Results are expressed as means ± SEM.

To further determine whether PLK inhibition enhances the sensitivity of GSCs to radiation, we subjected GSCs to different concentrations of volasertib for 24 hours followed by 2 Gy of radiation. Forty-eight hours after radiation, cleaved PARP was significantly greater in the combination treatment group than in the volasertib-treatment alone group (Figure 4A, Supplementary Figure 3). Consistent with the induction of apoptosis in the combination treatment group, there was a corresponding decreased expression of anti-apoptotic protein Bcl-xL and an increase expression of pro-apoptotic protein Bad. Similar findings were observed with respect to γ-H2AX, an index of DNA damage [24], which was significantly higher at 48 hours following radiation compared with the corresponding volasertib-treatment alone group. We further analyzed apoptosis by flow cytometry measurement of annexin V-propidium iodide staining. As shown in Figure 4B, there was no significant difference in apoptosis 48 hours after radiation compared with control; however, necrosis (as indicated by propidium iodide-positive staining) was higher in the radiation-treatment alone group. In contrast, apoptosis induction was significantly higher in the cells that received 100 nM volasertib plus 2 Gy of radiation than in the volasertib-treatment alone group. Taken together, these data suggest that the cytotoxic effects of combining volasertib and radiation are primarily mediated through apoptosis induction.

We performed the clonogenic (or colony-forming) assay, a widely used standard for evaluating radiation sensitivity of different cell lines *in vitro*, to determine the effects of volasertib on colony-forming ability and compared it with and without radiation. The average percentage of colony formation of post-irradiated GSCs was significantly lower compared to control, both in GSC23 (68.33% ± 0.95% versus 97.20% ± 1.10%, *P* < 0.01) and in GSC272 (96.13% ± 0.57% versus 88.9% ± 1.10%). Moreover, volasertib treatment at sub-IC_50_ doses (1 nM and 10 nM) resulted in a significant decrease in colony formation, which was further reduced by radiation treatment (Figure 4C). Together, our data demonstrated that the combination of volasertib and radiation synergistically inhibited colony formation *in vitro*.

### Combined volasertib and radiation synergistically inhibits tumor growth and prolongs median survival *in vivo*

**Figure 5:**
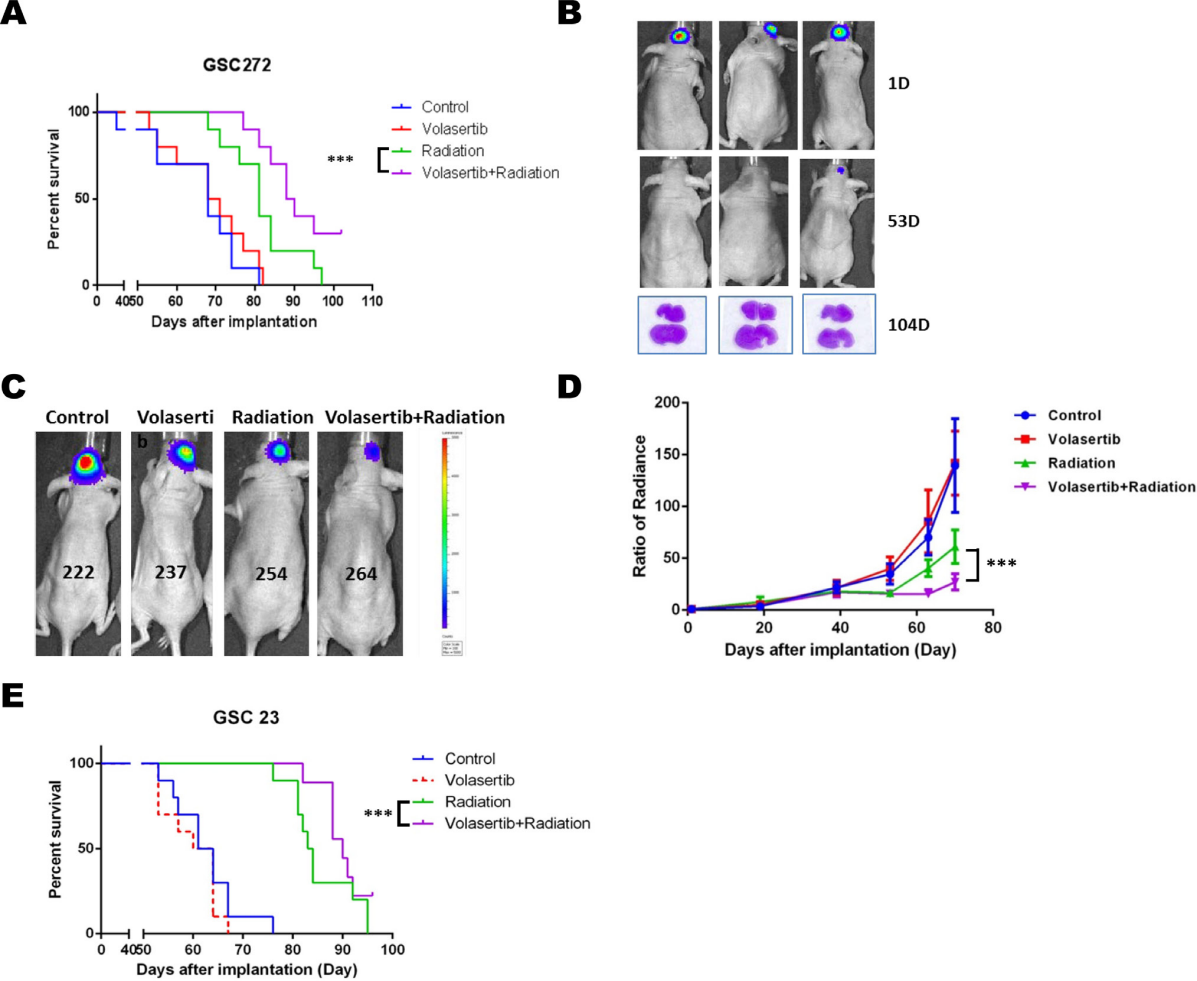
The combination of volasertib (10 mg/kg) and radiation (10 Gy) inhibited tumor growth and prolonged median survival in an intracranial mouse GSC xenograft model (**A**) Kaplan-Meier curve from the GSC272 model showed that the radiation group and combination group had significantly prolonged median survival compared with control and volasertib alone (*P* < 0.001 versus control). Notably, the combination treatment group had dramatically extended median survival compared with radiation treatment alone (*P* < 0.001 versus radiation), and the long-term survival of the combination group was 30% compared with 0% in other groups. (**B**) H&E staining of brain after the mice were sacrificed at 104 days after implantation showed no evidence of significant tumor progression suggesting that some tumors may have prolonged benefit from this combination. (**C**) Representative bioluminescent images of the GSC272 intracranial mouse xenograft model and the normalized average radiance (photons/s/cm^2^/sr) with various treatments and time points. (**D**) Radiation reduced tumor growth compared with control, which was further enhanced by combining radiation with volasertib (*P* < 0.001 versus radiation). Results are expressed as means ± SEM. (**E**) A similar response was seen in the GSC23 intracranial xenograft model, in which radiation treatment and combination treatment significantly prolonged median survival.

We further examined the combined effect of volasertib and radiation on intracranial xenograft models of GSCs. One week prior to initiating IR, volasertib (10 mg/kg) was administrated twice a week until the end of the experiment clinically relevant, fractionated IR (2.5 Gy × 4) was applied to mice 3 weeks after intracranial implantation of GSCs. As expected, mice that received GSC272 (Figure 5A) and GSC23 (Figure 5E) showed significantly improved median survival upon IR treatment alone compared to untreated controls, with median survival prolonged from 62.5 days to 83.5 days, and 68 days to 81 days respectively. Importantly, the combination of volasertib with IR treatment significantly improved median survival compared with IR alone in mice transplanted with GSC272 (89 days versus 68 days, *P* < 0.001) and with GSC23 (90 days versus 83.5 days, *P* < 0.001). Most importantly, 30% of the mice that received combination treatment demonstrated long-term survival (sacrificed at 104 days without moribund syndrome), whereas 0% of mice survived in the other treatment groups. In addition, H&E staining of combination-treated brains showed no evidence of significant tumor progression (Figure 5B), suggesting that some tumors may have prolonged benefit from this combination.

To further examine the effects of single treatment and combination treatment on tumor growth in mice, we expressed firefly luciferase in GSC272 to monitor tumor kinetics using bioluminescent imaging (Figure 5C and 5D). IR resulted in a strong decrease in tumor volume; however, tumor progression was eventually observed, by bioluminescence, at later time points, which suggests tumor recurrence. IR in combination with volasertib significantly sensitized IR-mediated tumor growth inhibition, with a significant delay in tumor volume growth compared with IR treatment alone (*P* < 0.001).

### Combination of volasertib and radiation synergistically inhibits tumor cell proliferation, induces DNA damage after radiation, and induces apoptosis *in vivo*

**Figure 6:**
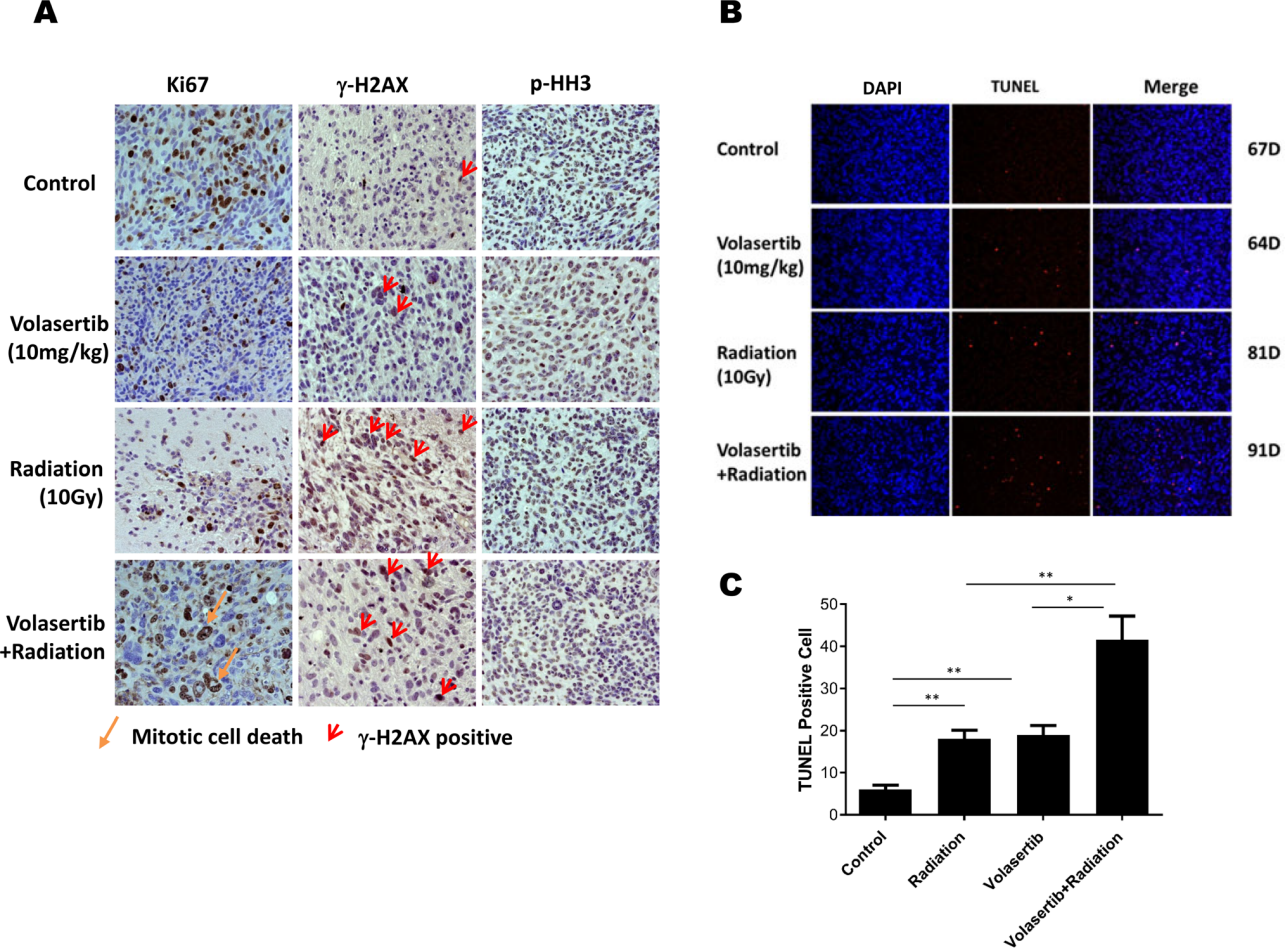
Combination of volasertib and radiation synergistically inhibits tumor cell proliferation, induces DNA damage and apoptosis *in vivo* (**A**) Immunohistochemical analysis of Ki-67, γ-H2AX, and p-HH3 in xenograft tumors that were treated with volasertib alone, radiation alone, and volasertib combined with radiation compared with untreated mice. A significant reduction in Ki-67 staining was observed in the volasertib, radiation, and combination treatment groups compared with control, indicating there were more mitotic cell deaths. The DNA damage marker γ-H2AX was enhanced in the radiation treatment group and the combination treatment group when the slides were stained the week following radiation. There were no significant p-HH3 changes among groups. (**B**) A TUNEL assay was performed to measure apoptosis in brain sections from GSC xenograft mice, with TUNEL in red and DAPI in blue. Arrows indicate examples of TUNEL-positive cells. (**C**) There were higher numbers of TUNEL-positive cells in the combined volasertib and radiation treatment group than in the control, volasertib-treatment alone, or radiation-treatment alone group. (^*^P < 0.05, ^**^P < 0.01).

Immunostaining of the proliferation marker Ki-67 was performed to identity proliferating cells in the xenografts subjected to different treatments. As shown in Figure 6A, the Ki-67-positive proliferating tumor cell population was significantly inhibited by volasertib, IR, and the combination compared with the control group. Moreover, significant mitotic cell death, as indicated by massive nuclear staining, was observed in the combination group. To determine whether volasertib could enhance IR-mediated DNA damage, γ-H_2_AX was evaluated 1 week following IR. DNA damage was significantly higher in the IR group compared with control and volasertib-treated groups, and this IR-mediated DNA damage was enhanced by treatment with volasertib. However, there was no dramatic difference in p-HH3 staining among the groups as shown in Figure 6A.

The aforementioned *in vitro* data suggest that the cytotoxic effects of combined volasertib and IR were synergistically mediated apoptosis induction; therefore, to determine whether similar effects would be observed in *in vivo* models, we assessed apoptosis staining by TUNEL. As shown in Figure 6B and 6C, TUNEL staining revealed significant apoptosis induction in the combination group compared with control and single-treatment groups. This pro-apoptotic effect of combined treatment correlated with prolonged median survival.

## DISCUSSION

Here, we show that PLK1 inhibitor volasertib synergistically enhances radiation efficacy *in vitro* and *in vivo* in GBM. The following lines of evidence support this statement. First, volasertib inhibited the viability of GSCs and induced G2/M arrest and apoptosis in a dose- and time-dependent manner. Second, GSCs, arrested in M phase by volasertib, were more sensitive to IR, as evidence by in inducing G2/M arrest, synergism in inducing apoptosis and inhibiting colony formation. Previous studies have shown that E1A and Ras transformed p53 null and wild type mouse embryonic fibroblasts do not undergo significant apoptosis after radiation and do not correlate to apoptotic genes Bcl2 in clonogenic survival assays [25]. However, our results shows that in, *in vivo* xenograft models the combination of volasertib and radiation significantly inhibited GSC tumor growth and prolonged median survival when compared with radiation alone. These differences could be attributed to organ specific cellular properties and variation in cell growth and culturing conditions. Here we show for the first time to our knowledge that high expressions of anti-apoptotic protein Bcl-xL was a biomarker highly correlated with volasertib resistance.

The treatment of GBM remains challenging despite meaningful progress, over the past two decades, in the molecular treatment of many other cancers, such as lung and breast. In recent years, significant progress in our understanding of the molecular pathogenesis of GBM has been made [8, 26–28]. Extensive profiling of the GBM genome has identified multiple activated signaling pathways that play a central role in cancer cell growth, survival, motility, and metabolism, which represent potential therapeutic targets [8, 26–28]. However, the development of targeted therapy is complicated by the complexity and redundancy of these signaling pathways.

The presence of self-renewing GSCs is thought to be responsible for tumor initiation, heterogeneity, and resistance to standard therapies. These GSCs closely resemble the parent tumor both genotypically and phenotypically, thus making the use of GSCs a desirable approach for developing more effective therapeutic strategies. The Brain Tumor Center at MD Anderson Cancer Center has been collecting GSCs from patient tumor samples and profiling them by a number of “omics” techniques [29, 30]. In this study, we used HTS to identify the drug sensitivities of a panel of GSC lines to a panel of compounds in PKIS, and we identified sensitivity of GSCs to PLK1 inhibitors. Similar results were reported by another group, who used high-content imaging-based screening assays and who observed that GSCs were acutely susceptible to proliferative disruption by PLK1 inhibitors [31].

PLKs are a group of highly conserved serine/threonine kinases with various key regulatory functions during cell cycle progression, and PLK1 is the best characterized cancer target in the PLK family [10, 13, 16]. PLK1 is overexpressed in several tumor types, including breast cancer, non-small cell lung cancer (NSCLC), and colorectal cancer, and elevated PLK1 has also been correlated with poor prognosis in NSCLC and breast cancer patients [13, 32, 33]. Nonetheless, the promising effects of PLK1 inhibition in GBM remain poorly developed [23, 34, 35]. To further validate the HTS results, we used volasertib to investigate the potential therapeutic application of PLK1 inhibitors in GBM. Volasertib, a second generation dihydropterinone derivative, is a potent ATP-competitive selective inhibitor of PLK1, and it also inhibits PLK2 and PLK3 [11]. It has demonstrated broad antitumor activity with a high volume of distribution, indicating good tissue penetration, and a long terminal half-life in preclinical studies [36]. Several phase I, II, and III clinical trials have evaluated the efficacy of volasertib in treating leukemia, lung cancer, and pancreatic cancer and have shown encouraging results [11, 12, 37–39]. However, few reports have considered the use of volasertib in GBM. In the current study, we found that volasertib potently inhibited the proliferation of a panel of GSC lines with nanomolar IC_50_. Data from the RPPA studies, verified with Western blotting, determined that high expression of Bcl-xL was associated with resistance to volasertib in the cell lines tested. Consistent with previous studies, volasertib promoted mitotic exit delay, which resulted in G2/M arrest and subsequent induction of apoptosis [22, 23]. Loss of p53 and/or p21^Cip1/CDKN1A^ renders cancer cells susceptible to PLK1 inhibition [31, 40]. Further studies will be necessary to determine if tumors with p53 mutations, loss of CDKN1A, or other molecular markers are more susceptible to this combination strategy.

Acquired resistance to therapy is a common problem in patients with GBM. Of the three main therapies for GBM—surgical resection, radiation therapy, and chemotherapy—radiation remains the most relatively efficacious therapy for primary brain tumors [1]. However, many patients have disease refractory to radiation therapy, and this radioresistance ultimately leads to tumor recurrence. Therefore, there is an urgent need to develop strategies to radiosensitize GBM tumor cells. Hence, we tested the efficacy of the combination of volasertib with radiation in preclinical models, as a prerequisite to future clinical studies. The *in vitro* data in this study showed that the combination of volasertib with radiation improved radiation efficacy in inhibiting colony formation and inducing apoptosis. Here, we show, for the first time, that combination treatment significantly delayed tumor growth in mice implanted with GSC23 or GSC272, and significantly prolonged median survival by inhibiting tumor growth and inducing apoptosis.

One potential limitation of the current study is that volasertib treatment alone showed no effects on inhibiting *in vivo* tumor growth or prolonging median survival. This may be attributed to poor biodistribution of volasertib within the brain, due to a suboptimal dose and/or a relatively intact blood-brain barrier. In the limited amount of literature reporting on the use of PLK1 inhibitors, the PLK1 inhibitors with significant antitumor effects in GBM have been GSK461364A at 100 mg/kg [22] and BI2536 at 50 mg/kg [34]. The dose of volasertib that we chose for this study, 10 mg/kg, may not have been sufficient as a monotherapy in our complex *in vivo* model. However, radiotherapy can increase blood-brain barrier permeability and improve drug delivery to brain tumors [41, 42]. Therefore, in our study, radiation therapy may have increased the brain biodistribution of 10 mg/kg volasertib, leading to synergistic enhancement in radiation therapy-mediated tumor growth inhibition and median survival prolongation. Moreover, microenvironmental factors including hypoxia, molecular heterogeneity, and tumor invasion may also limit the *in vivo* efficacy of volasertib alone [2, 43]. Accordingly, we cannot exclude the possibility that the beneficial effects of volasertib observed *in vitro* might be less evident *in vivo*, and further optimization of this combined approach is needed to fully realize the benefit.

Taken together, the findings of our study both confirm and expand on previous *in vitro* and *in vivo* studies by demonstrating that volasertib inhibits tumor proliferation, induces G2/M arrest, and induces apoptosis by sensitizing GSCs to radiation to synergistically delay tumor growth and prolong median survival. This study has convincingly shown that targeting PLK1 with volasertib, in combination with radiation therapy, may be a novel strategy to overcome resistance in the treatment of patients with GBM. Additional studies will be required to investigate the mechanisms of the synergistic effect of this promising combination regimen and to further optimize the safety, feasibility, and clinical effectiveness of this therapy.

## MATERIALS AND METHODS

### GSC isolation and cell culture

GSC lines were isolated from brain tumor specimens at The University of Texas MD Anderson Cancer Center, Houston, TX. The Institutional Review Board of MD Anderson Cancer Center approved acquisition of these cell lines from patients, who provided informed consent. Glioma stem cell development is funded by the MD Anderson Brain Cancer SPORE supported by P50CA127001. GSCs were maintained in DMEM/F12 medium supplemented with B27 supplement (Invitrogen), 20 ng/ml epidermal growth factor (EGF), and 20 ng/ml fibroblast growth factor (FGF) at 37°C in a humidified atmosphere of 5% CO_2_ and 95% air [5, 30]. GSCs were tested and authenticated by DNA typing at the MD Anderson Cancer Center Cell Line Characterization Core and were subsequently verified for our study.

### High-throughput screening

GlaxoSmithKline provided the PKIS with 357 compounds, including the 21 most highly active compounds. A panel of GSC cell lines, which have been well characterized for their protein and gene expression, was screened in 384-well plates at a density of 1000 cells/well with 16 serially diluted drug concentrations. On the same day, 50 nl of each compound was transferred into cell wells using the Tecan Evo200 robotic system. Five days after drug treatment, cell viability was determined by using the CellTiter-Blue cell viability assay and compared with values from vehicle control wells to calculate the half-maximal inhibitory concentration, IC_50_. Each cell line was screened in duplicate. The quality of the assay was estimated by calculating a Z′-factor. Average Z′ value for all screens was 0.7 ± 0.5. A separate set of compounds, containing doxorubicin and molecularly targeted agents to tumor growth factor beta (TGF-β), signal transducer and activator of transcription 3 (STAT3), phosphoinositide 3-kinase (PI3K), and epidermal growth factor receptor (EGFR), was used as the positive control plate to compare drug efficacy.

### Cell cycle and apoptosis analysis by flow cytometry

After drug treatment, GSCs were pelleted by centrifugation and dissociated with Accutase cell detachment solution (Sigma). Cells were collected, washed with PBS, and fixed with ice-cold 70% ethanol for at least 1 hour. Then, cells were washed twice in PBS, treated for 30 minutes at 37°C with PI/RNase Staining Buffer (BD Biosciences), and analyzed using a FACScan flow cytometer (Becton-Dickinson) to determine subG1 (apoptosis), G1, S, and G2/M cell cycle distribution. Apoptosis was detected using the BD Annexin V FITC Assay on the BD FACSVerse System (BD Biosciences).

### Clonogenic formation assay

For evaluation of clonogenic formation, post-treatment GSCs were seeded at 3–5 cells per well in triplicate 96-well plates for 3 weeks [29]. Wells with neurospheres (>100 micron M) were counted as positive and wells without spheres as negative. The percentages of positive neurospheres in each plate were compared among different treatment groups.

### Western blot analysis and reverse-phase protein array (RPPA)

Cells were lysed in an ice-cold RIPA lysis buffer containing a cocktail of proteinase inhibitors and phosphatase inhibitors. The protein concentration in the supernatant was determined using the BCA protein assay (Pierce Chemical). Samples were subjected to 8% to 15% SDS-PAGE, and the separated proteins were electrophoretically transferred to PVDF membranes. Blots were incubated with the primary antibodies, and then incubated with horseradish peroxidase-linked secondary anti-rabbit or anti-mouse antibodies (GE). GSC protein lysate samples were probed with 279 validated primary antibodies for the analysis at the MD Anderson Functional Proteomics Reverse Phase Protein Array (RPPA) Core facility. (https://www.mdanderson.org/education-and-research/resources-for-professionals/scientific-resources/core-facilities-and-services/functional-proteomics-rppa-core/index.html).

### Xenograft models and treatment

GSCs were intracranially implanted, using the guide-screw system, into 4- to 6-week-old female nude mice [44]. One week after guide screw implantation, 500,000 cells were intracranially injected into each mouse, and the mice were randomly distributed to receive different vehicle control, volasertib (10 mg/kg), 2.5-Gy IR, or volasertib (10 mg/kg) plus 2.5-Gy IR. A minimum of 10 mice was used in each treatment group to generate survival curves. For *in vivo* bioluminescent imaging, GSCs were engineered to express luciferase by transducing GSCs with pCignal lenti-CMV-luc viral particles (SABiosciences). Kinetics of tumor growth was monitored by IVIS 200 system bioluminescent imaging. Two weeks after GSC implantation and one week before IR, volasertib (10 mg/kg) was administrated twice a week by oral gavage until the end of the experiment. The radiation treatment involved four cycles of 2.5-Gy IR, on four consecutive days. IR was delivered using a ^60^Co tele-therapy unit and a custom gig with validated dosimetry. Mice with neurological symptoms (i.e., hydrocephalus, seizures, inactivity, and/or ataxia) or that were moribund were euthanized. Brains were fixed in formalin, stained with H&E to confirm the presence of tumor, and subjected to immunohistochemical analysis. All animal procedures were reviewed and approved by the Institutional Animal Care and Use Committee at MD Anderson Cancer Center.

### Immunohistochemistry and TUNEL assay

Paraffin sections from xenografts were used for immunohistochemical analysis. The slides were deparaffinized and subjected to graded rehydration. After an antigen retrieval step (citrate buffer, pH 6.0) and blocking in 5% serum, the slides were incubated with the primary antibodies overnight at 4°C. After washing in PBS with Tween 20, primary antibody reactions were detected using the Vectastain ABC kit (Vector Laboratories) with the appropriate secondary antibody. The apoptosis assay in mouse tissues was performed according to the manufacturer's protocol (*In Situ* Cell Death Detection Kit, TMR red; Roche).

### Data analysis

All data are expressed as means ± SEM. One-way repeated measures analysis of variance (ANOVA) and two-way ANOVA followed by post-hoc multiple comparisons were used to establish significant differences between groups. The Kaplan-Meier curves were compared using the log rank test. After log rank comparisons, multiple comparisons were performed using the Tukey test. A value of *P* ≤ 0.05 was considered statistically significant.

We used a Wilcoxon Test to calculate *p*-values to compare compounds IC50 with TCGA subtypes, and RPPA protein expression between sensitive and resistant GSCs. We ranked RPPA proteins according to the significance comparing sensitive and resistant GSCs using Limma [45] and the R language [46]. Proteins were ranked and selected according to *p*-value.

## SUPPLEMENTARY MATERIALS FIGURES AND TABLES


