# Supplementary material for: The polo-like kinase 1 inhibitor volasertib synergistically increases radiation efficacy in glioma stem cells
Source: Oncotarget. 2018 Jan 8;9(12):10497–509. doi: 10.18632/oncotarget.24041 (PMC5828226; doi:10.18632/oncotarget.24041)
Supplement: Supplementary file 1 [file oncotarget-09-10497-s001.pdf]

The polo-like kinase 1 inhibitor volasertib synergistically increases radiation efficacy in glioma stem cells

SUPPLEMENTARY MATERIALS

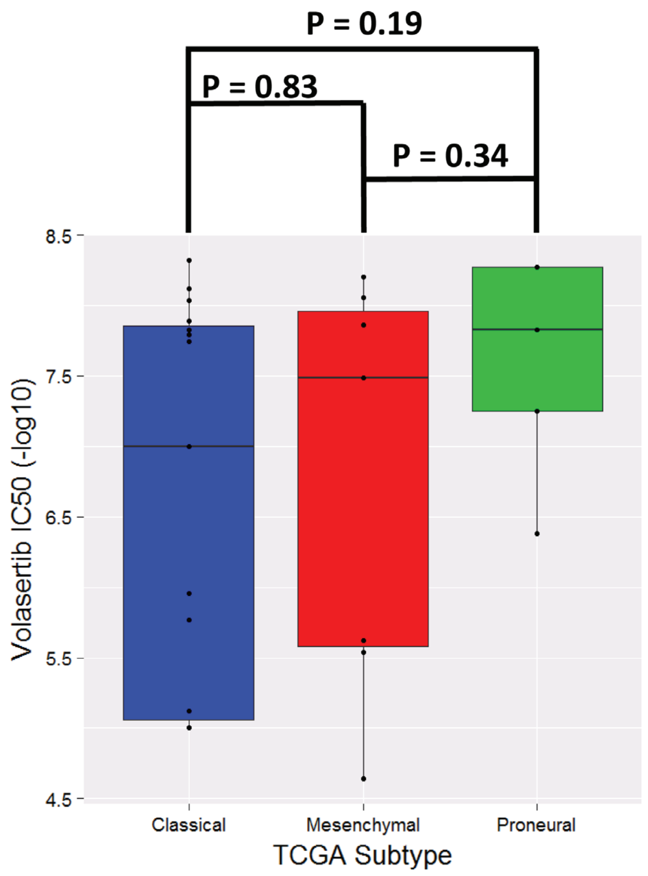

Supplementary Figure 1: TCGA subtype comparison for Volasertib response. *P*-values were calculated using a Wilcoxon test.

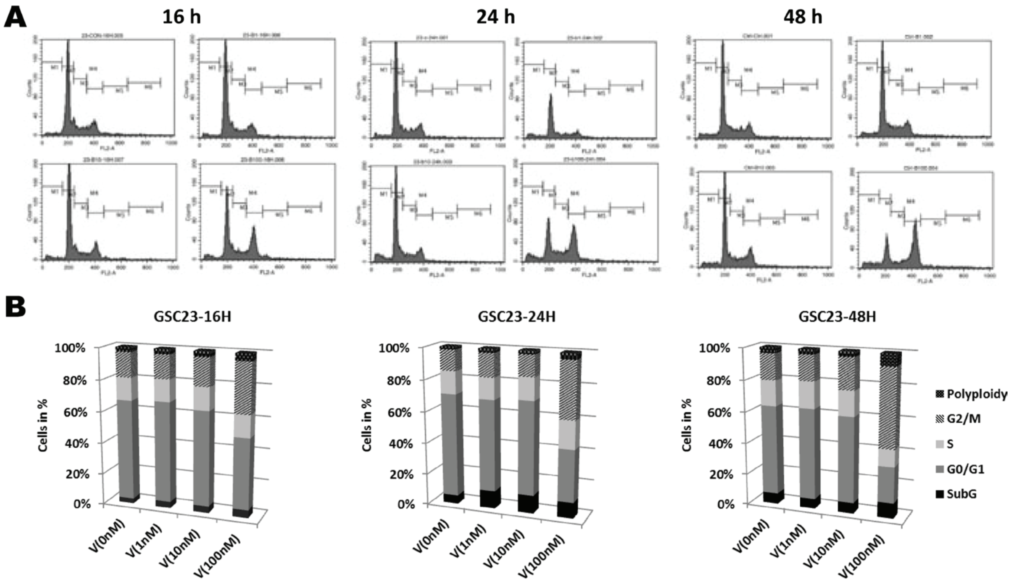

Supplementary Figure 2: Volasertib arrest G2/M phase in GSC23. (**A–B**) GSCs were treated with volaserib time dependent, and then cell cycle distribution was analyzed time points.

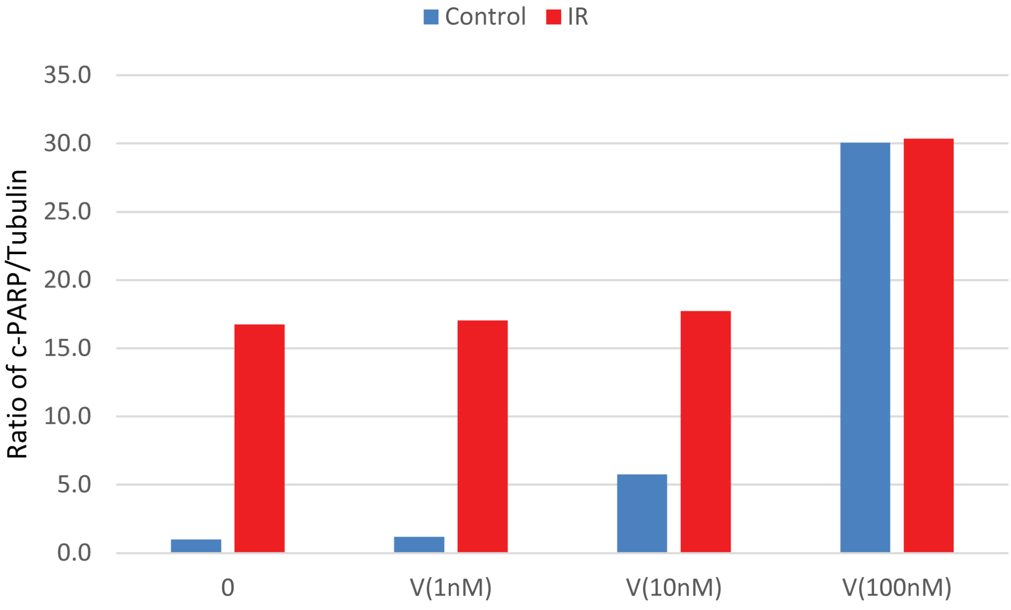

Supplementary Figure 3: PLK1 inhibition increased cleaved PARP expression with or without radiation. Quantified the c-PARP and total tubulin in Figure 4A by using Image-J software.

Supplementary Table 1: IC_{50} values for GSK compounds (values at µM level)

	GSC20	GSC11	GSC8-11	GSC23	GSC7-2	GSC231	GSC28	GSC6-27	GSC272	GSC17	GSC280	
**GSK1007102B**	0.561	0.219	0.049	1.293	1.699	2.173	0.774	4.244	1.579	2.073	2.232	
**GSK1173862A**	5.489	1.496	2.194	2.025	3.381	4.047	5.353	6.214	2.258	3.868	3.319	
**GSK1220512A**	2.974	0.907	2.223	2.778	3.337	2.933	4.231	2.974	2.191	3.119	2.287	
**GSK2110236A**	2.568	0.734	1.075	2.065	2.528	1.790	3.962	4.738	2.098	1.738	1.545	IC50 < 2 µM
**GSK2186269A**	0.486	0.269	0.114	0.071	0.131	1.549	1.212	2.537	0.262	1.418	1.637	
**GSK2213727A**	5.145	0.797	2.531	2.345	3.755	2.275	4.497	6.202	3.530	2.071	2.252	
**GSK317315A**	8.458	0.873	1.396	3.484	3.852	3.413	4.729	7.470	2.692	3.560	3.751	
**GSK579289A**	2.527	0.246	1.113	1.607	2.146	1.671	3.979	6.147	1.049	0.985	3.093	
**GSK978744A**	10.380	6.420	6.528	6.217	6.164	6.871	16.068	11.267	11.803	16.175	8.641	
**GW301789X**	19.518	8.255	15.209	18.427	9.162	12.644	62.769	16.729	46.263	30.282	17.282	
**GW416981X**	5.841	3.062	3.078	2.366	2.763	4.921	8.128	7.524	3.987	5.644	6.584	
**GW778894X**	1.262	0.310	0.517	0.044	0.023	1.319	2.381	4.093	0.421	0.891	1.085	
**GW779439X**	0.200	0.143	0.056	0.174	0.509	0.561	0.958	3.199	0.484	0.503	0.713	
**GW780056X**	0.188	1.344	0.037	0.021	1.383	0.349	1.451	3.324	0.354	0.481	0.452	
**GW801372X**	0.500	0.346	0.237	0.180	1.075	2.011	2.954	4.047	1.007	1.603	1.428	
**GW806290X**	1.824	0.259	0.142	0.108	0.121	1.831	3.712	5.996	1.027	1.430	1.461	
**GW806742X**	1.880	0.691	0.618	0.424	0.696	2.703	3.747	3.253	2.197	2.929	2.332	
**GW809897X**	6.055	1.236	1.615	1.433	3.210	2.672	2.648	7.272	3.981	2.644	1.623	
**GW810576X**	1.134	0.167	0.103	0.061	0.699	1.115	1.398	2.857	0.759	0.876	0.740	
**GW852849X**	9.894	4.275	4.911	3.703	5.732	4.257	11.593	9.601	3.501	3.730	5.470	
**GW856804X**	8.284	8.288	14.745	8.390	24.250	2.527	17.292	10.835	4.973	40.701	2.261	

Supplementary Table 2: Pearson correlation between IC_{50} (-log10) and AUC for the GSK compounds

	Correlation		
**Compound**	**Coefficient**	***p*-value**	**Significant (*p*-value < 0.05)**
GW780056X	–0.4798633	0.135247162	No
GW810576X	–0.5915706	0.055233791	Yes (barely)
GW301789X	0.4801002	0.135026555	No
GW801372X	–0.5491391	0.080183407	No
GW778894	–0.7898853	0.00382325	Yes
GSK579289A	–0.8939478	0.000206523	Yes
GSK2110236A	–0.6657622	0.025341072	Yes
GSK2186269A	–0.5031335	0.114660815	No
GSK1173862A	–0.5970238	0.052470668	Yes (barely)
GW806290X	–0.6308306	0.037424277	Yes
GW852849X	–0.8795241	0.00035877	Yes
GSK317315A	–0.5973541	0.05230638	Yes (barely)
GSK1220512A	–0.5613979	0.07232807	No
GW779439X	–0.5761049	0.063606607	No
GSK2213727	–0.5989852	0.0515004	Yes (barely)
GSK1007102B	–0.8952773	0.000195512	Yes
GW856804X	0.1025895	0.764059893	No
GW806742X	–0.7127946	0.013818483	Yes
GSK978744A	–0.4139682	0.205613711	No
GW809897X	–0.8746517	0.000425719	Yes
GW416981X	–0.8238754	0.001821099	Yes

Supplementary Table 3: Wilcoxon test *p*-values comparing TCGA subtypes for the GSK compounds

Compound	Classical vs Mesenchymal	Significant (*p*-value < 0.05)
GSK1007102B	0.3524	No
GSK1173862A	0.4762	No
GSK1220512A	0.3923	No
GSK2110236A	0.4762	No
GSK2186269A	0.9143	No
GSK2213727A	0.6095	No
GSK317315A	0.6095	No
GSK579289A	0.9143	No
GSK978744A	0.0190	Yes
GW301789X	0.0095	Yes
GW416981X	0.3524	No
GW778894X	0.6095	No
GW779439X	0.9143	No
GW780056X	0.9143	No
GW801372X	0.9143	No
GW806290X	0.6095	No
GW806742X	0.3524	No
GW809897X	0.4762	No
GW810576X	0.2571	No
GW852849X	0.9143	No
GW856804X	0.6095	No
